# Phenolic Profile, Antioxidant, Antidiabetic, and Antigout Potential of Stem Extracts of Four Sweet Cherry Cultivars

**DOI:** 10.1155/2023/8535139

**Published:** 2023-05-05

**Authors:** Younes Aqil, Souad El Hajjaji, Walid Belmaghraoui, Yassine Mourabit, Douae Taha, Mohammed Merae Alshahrani, Ahmed Abdullah Al Awadh, Abdelhakim Bouyahya, Issam Gaamoussi, Ilhame Bourais

**Affiliations:** ^1^Laboratory of Spectroscopy, Molecular Modelling Materials, Nanomaterials, Water and Environment Faculty of Sciences, Mohammed V University in Rabat, Rabat, Morocco; ^2^Department of Clinical Laboratory Sciences, Faculty of Applied Medical Sciences, Najran University, Najran 61441, Saudi Arabia; ^3^Laboratory of Human Pathologies Biology, Department of Biology, Faculty of Sciences, Mohammed V University in Rabat, Rabat, Morocco; ^4^Laboratory of Research and Development, AROMI Sarl, 197 BD, Casablanca, Morocco

## Abstract

In order to highlight the activities of bioactive compounds present in the stem of sweet cherries, four different cultivars (*Van*, *Burlat*, *Napoleon*, and *Cœur pigeon*) were collected in Sefrou city in Morocco and were studied. Several assays were performed for this purpose, such as the quantification of phenolic compounds (TPC, TFC, and CTC) and the evaluation of the antioxidant activity using DPPH, ABTS, and FRAP assays. The phenolic profile of each extract was characterized by UHPLC-DAD/MS analysis. The antidiabetic (*α*-amylase inhibition) and antigout (xanthine oxidase inhibition) activities were also investigated. The results showed high levels of phenolic compounds, with the values of 340 ± 12.06, 244 ± 10.20, 232 ± 5.07, and 19 ± 3.10 mg gallic acid equivalent/g extract for the cultivars *Napoleon*, *Coeur de pigeon*, Van, and *Burlat*, respectively. According to the same order, the flavonoids showed amounts of 34.31 ± 2.08, 23.75 ± 1.02, 24.37 ± 1.20, and 23.31 ± 0.90 mg (rutin equivalent) RE/g extract. These values were correlated with the results of the antioxidant assays, where the *Napoleon* cultivar proved to be the most potent using the DPPH (IC_50_ = 2.51 *µ*g/mL) and ABTS (IC_50_ = 55.38 *µ*g/mL) assays. The phenolic profile of each extract resulted in the identification of twenty-two compounds belonging to five distinct groups. The major phenolic compounds identified were sakuranetin and dihydrowgonin with their glucosides. Antidiabetic activity assays showed that only stem extracts from *Burlat* and *Napoleon* cultivars were able to inhibit the *α*-amylase enzyme with values of 85.57 ± 1.09% and 68.01 ± 3.52%, respectively. All stem extracts proved their ability to inhibit the xanthine oxidase enzyme which is directly linked to the gout disease, with a high value for *Van* cultivar (40.63 ± 2.37%). These new findings could provide new opportunities for the valorization of cherry stems for the pharmaceutical application of their active phytochemicals.

## 1. Introduction

In recent years, many research studies proved the importance of fruits and vegetables in promoting human health for their unique beneficial nutrients and antioxidants, encouraging their increasing consumption both nationally and internationally [1–3]. Cherries, from the Rosaceae family, Prunodea subfamily, are one of the most commercially important species in the world and the most consumed. *Prunus avium* L. (sweet cherry) is geographically distributed worldwide, with greatest predominance in temperate climates, encompassing much of Europe (Mediterranean and Central), the Near and Far East, the southern Australia and New Zealand, North Africa, and the temperate zones of the American continent [4].

In Morocco, sweet cherry was introduced before 1920 by the French protectorate, and it was extended to the regions of the Moroccan Middle Atlas. Actually, the cultivation covers 4000 ha of the area with the production of about 14,100 tons each year, and the most popular sweet cherry cultivars are “*Bigarreau Van*,” “*Bigarreau Burlat*,” “*Bigarreau Napoleon*,” and “*Cœur pigeon*,” [5, 6].

Sweet cherry fruits are one of the most studied by the scientific community for their nutritional and bioactive properties. Many beneficial effects have been recognized, especially the control of diabetes, the prevention of cardiovascular disease, cancer, gout, and other diseases related to oxidative stress [7].

It is noted that little research was conducted on sweet cherry stems, as well as their chemical composition and bioactive properties [5, 7–14].

Sweet cherries are also renowned for their use by food industries for the manufacture of jams, jellies, compotes, and several types of beverages, subsequently generating important waste in the form of stems or kernels. In this context, this study aims to investigate the total content of phenolic compounds, flavonoids, and tannins of cherry stem extracts from different cultivars, as well as their antioxidant properties using DPPH, ABTS, and FRAP methods, with the characterization of the phenolic compounds present in the extracts by the UHPLC-DAD/MS technique [12]. Furthermore, the antidiabetic and antigout activities were evaluated with a comparative approach of four cultivars. Sweet cherry cultivars have not been analyzed in this sense until now.

## 2. Materials and Methods

### 2.1. Raw Materials

The harvest area is located in the Middle Atlas region, specifically Laanoceur (Sefrou). The “Laanoceur” locality is known for its continental climate with cold winters and hot summers, with annual average rainfall varying between 400 and 600 mm and an annual average temperature of 10.6°C [5].

**Figure 1:**
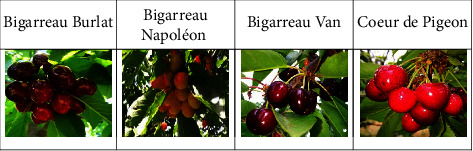
Pictures of the different cherry fruits varieties before harvest.

Fruit from four sweet cherry (*Prunus avium*) cultivars (*Burlat*, *Napoleon*, *Coeur de pigeon*, and *Van*) were collected at the optimum fruiting period, based on fruit maturity and bright red color (Figure 1). The stems were removed from the cherry fruits, washed with distilled water, and dried in the shade at room temperature. Then, these stems were ground into a fine powder and stored in hermetic bags at 4°C until further use.

### 2.2. Extraction Method

The hydromethanolic extracts were obtained from the powdered stems of each cultivar. Each of these stems (5 g) was extracted using an ultrasonic sonotrode (Hielscher UP100H) applying 30 kHz for 30 minutes using 50 mL of methanol/water (80 : 20, v/v) as solvent and then filtered using Whatman No. 4 paper. The extracts were then concentrated using a vacuum rotary evaporator (R-100, BUCHI) at 40–55°C under vacuum, and the dried residues were stored in Eppendorf tubes at −4°C for further studies. Yields obtained from the starting material were 17.25 ± 0.90 g, 16.90 ± 0.41 g, 18.34 ± 1.01 g, and 22.18 ± 1.12 g for *Burlat*, *Napoleon*, *Coeur de pigeon*, and *Van* cultivars, respectively. Extractions were performed in triplicate.

### 2.3. Determination of the Total Phenolic Content

The phenol content test was performed using the Folin–Ciocalteu technique as detailed in previous investigations [15, 16].

Thus, 0.5 mL of the sample solution was mixed with 2.5 mL of Folin–Ciocalteu reagent diluted with distilled water in a ratio of 1 : 10, and then 4 mL of Na_2_CO_3_ (7.5%, w/v) was added. Afterwards, a 45°C water bath was used to heat the mixture for half an hour and absorbance measurements were made at 765 nm using a UV-Vis spectrophotometer in comparison with the blank solution. Under the same conditions, the standard curve of gallic acid was obtained over a concentration range of 0–300 mg/L. The values of phenolic contents were expressed as gallic acid equivalent (mg GAE/g extract). The test was carried out in triplicate for all the samples as well as for the standards and the blank (distilled water).

### 2.4. Determination of the Flavonoids Content

The flavonoid content was determined according to the method described in the literature with slight modifications [15, 17]. The total flavonoid content was determined using 0.50 mL of each extract stock solution (1 mg/mL) and each dilution of rutin standard solution (10–100 *μ*g/mL) taken separately in test tubes. To each test tube, 1.50 mL methanol, 0.10 mL aluminum chloride solution, 0.10 mL potassium acetate solution, and 2.80 mL distilled water were added and shaken. Blank samples for all extracts and standard rutin dilutions were prepared similarly by replacing the aluminum chloride solution with distilled water. All the prepared solutions were filtered on Whatman No. 1 filter paper before measuring their absorbance at 510 nm against the appropriate blank. From a rutin calibration curve, the total flavonoid content was calculated, and the result was expressed in mg of rutin equivalent per gram of the dry extract (mg RE/g extract). The test was carried out in triplicate for all the samples as well as for the standards and the blank.

### 2.5. Determination of the Proanthocyanidins Content

The CTC content was performed as described in the previous research studies [15, 18]. Thus, 3 mL of 4% vanillin-methanol solution was added to 0.05 mL of the extract with the addition of 1.5 mL hydrochloric acid. Subsequently, the mixture is left to stand for 15 minutes. Absorbance was measured at a wavelength of 500 nm, and the results were reported in mg catechin equivalent (CE)/g extract. The assay was carried out in triplicate for all the samples as well as for the standards and the blank.

### 2.6. Antioxidant Activity

The assays were carried out in triplicate for all the samples as well as for the standards and blank as indicated below:

#### 2.6.1. Free Radical Scavenging Activity

The free radical scavenging activity of the extracts was measured by 1.1-diphenyl-2-picryl-hydrazyl (DPPH) [15, 19]. Thus, 0.5 mL of a 0.2 mM DPPH solution was mixed with 2.5 mL of the extract. The obtained solution was left at 25°C for about 30 minutes, and absorbance was recorded at 517 nm against blank samples. The radical-scavenging activity (RSA) was expressed as a percentage of discoloration. Equation (1) was used to calculate percent inhibition from the obtained absorbance:

(1) $$\begin{array}{c} \%\;RSA=\frac{A_{D}-A_{E}}{A_{D}}\times 100\text{,} \end{array}$$

where *A*_*D*_ is the recorded value of the blank sample and *A*_*E*_ is the value of the test solution. *A*_*E*_ was determined as the difference between the value of the test solution and the obtained value of its blank.

The IC_50_ value was determined from the graph of the scavenging activity against a range of extract concentrations and is defined as the concentration of antioxidants required to decrease the initial concentration of DPPH radicals by 50%.

#### 2.6.2. ABTS Radical Cation Decolorization Assay

The ABTS radical cation decolorization test was performed as described in [15, 20]. A stock solution of 2 mM ABTS was mixed with 70 mM potassium persulphate stock solution (v/v), and the obtained solution was stored in the dark for 24 h at 25°C. Methanol was carefully added to reach 0.7 ± 0.2 units at 734 nm. Afterwards, 2 mL of the resulting solution was added to 200 *µ*L of the extract, the whole solution was mixed, and the absorbance was recorded at 734 nm at an interval of 1 minute. The results were determined using equation (2) and reported as a percentage of the inhibition of free radical scavenging relative to the blank solution.

(2) $$\begin{array}{c} ABTS\;radical\;scavenging\;activity\;\left(\%\right)=\left(1-Abs\;sample-Abs\;blank\right)\times 100\text{,} \end{array}$$

where Abs blank is the value of the blank solution, and Abs sample is the value of the test sample.

#### 2.6.3. Evaluation of Ferric Reducing Antioxidant Power (FRAP)

This method is designed around the capacity of the extract to reduce ferric ion (Fe^3+^) to the corresponding ferrous ion (Fe^2+^). The remaining Fe^2+^ ions form a blue complex with the reagent 2,4,6-tris(2-pyridyl)-s-triazine (TPTZ), which reaches the absorption maximum at 700 nm. The assessment of the ferric reducing antioxidant power (the FRAP test) was carried out as described by Ouerghemmi and coauthors in [21].

In brief, a volume of 0.25 mL of each sample (1 mg/mL) was added to 1.25 mL of sodium dihydrogen phosphate buffer (0.2 M, pH 6.6) with 1.25 mL of potassium ferricyanide solution (1%), and the mixture was incubated at 50°C for 20 min, followed by the addition of 1.25 mL of trichloroacetic acid (10%), and after centrifugation at 3000 tr/min for 10 min, the supernatant of the solution (1.25 mL) was added to 0.25 mL of iron (III) Chloride (0.1%). The absorbance was measured after a 30 min incubation period at room temperature at 700 nm. An increasing absorbance value means a high reduction capacity. Trolox was used as a standard for the calibration curve (20–400 *μ*g/mL). Finally, the reducing power was represented as Trolox equivalent (*µ*g·TE/g of extract).

### 2.7. Phytochemical Analysis by UHPLC-DAD/MS

The extracts were analyzed using a Hewlett-Packard 1100 chromatograph (Agilent Technologies) with a quaternary pump and a diode array detector (DAD) coupled to an HP Chem Station (rev. A.05.04) data-processing station. A Waters Spherisorb C18, 5 *µ*m (2.1 mm × 150 mm) column thermostated at 35°C was used. The solvents used were (A) 0.1% formic acid in water and (B) acetonitrile. The elution gradient established was isocratic 15% for 5 min, 15% B to 20% B over 5 min, 20–25% B for 10 min, 25–35% B for 10 min, 35–50% for 10 min, and reequilibrate the column using a flow rate of 0.5 mL/min. The double online detection was carried out in the DAD using 280 nm and 370 nm as preferred wavelengths and in a mass spectrometer (MS) connected to the HPLC system via the DAD cell outlet.

MS detection was performed in an API 3200 Qtrap (Applied Biosystems, Darmstadt, Germany) equipped with an ESI source and a triple quadrupole-ion trap mass analyzer controlled by the Analyst 5.1 software. Zero-grade air served as the nebulizer gas (30 psi) and turbo gas for solvent drying (400°C, 40 psi). Nitrogen served as the curtain (20 psi) and collision gas (medium). The Quadrupols were set to unit resolution. The ion spray voltage was set at −4500 V in the negative mode.

### 2.8. Assessment of Bioactive Properties

#### 2.8.1. Antidiabetic Activity (the *α*-Amylase Inhibitory Activity)

The *α*-amylase inhibitory activity was performed according to the protocol of Kusano and colleagues with some modifications [22].

The substrate was prepared by dissolving 200 mg of starch in 25 mL of sodium hydroxide (0.4 M) by heating at 100°C for 5 min. After cooling, the pH was adjusted to 7.0 and the volume was made up to 100 mL with distilled water. Acarbose was used as a positive control. Sample solutions were prepared by dissolving each sample in phosphate buffer (pH 6.5) to obtain 1 mg/mL solutions. 100 *μ*L of *α*-amylase 3 U/mL (20 mM phosphate buffer with 6.7 mM NaCl, pH 6.9) was preincubated at 37°C for 15 min with 100 *μ*L of acarbose at different concentrations and extract solutions, followed by 500 *μ*L of the substrate solution and incubated at 37°C for 15 min. The reaction was terminated by the addition of 400 *μ*L of HCl (0.1 M), followed by the addition of 400 *μ*L of iodine reagent (2.5 mM). The absorbance was measured at 630 nm. The inhibition percentage of each sample was calculated using the following equation:

(3) $$\begin{array}{c} PI\left(\%\right)=100-\left[\frac{\left(B-A\right)-\left(D-C\right)}{\left(B-A\right)}\times 100\right]\text{.} \end{array}$$

*A* is the absorbance of the enzyme with the substrate. *B* is the absorbance of the phosphate buffer solution with the substrate. *C* is the absorbance of the enzyme with the inhibitor and the substrate. *D* is the absorbance of the phosphate buffer solution with the substrate and enzyme.

#### 2.8.2. Assessment of the Xanthine Oxidase Inhibitory Activity

The inhibitory activity of individual extracts towards xanthine oxidase (XOD) was assessed by adjusting the method used by EL Euch and coauthors with a slight modification [23]. In this method, xanthine oxidase was used as the enzyme catalysing the formation of reactive oxygen species (ROS) and produces uric acid. In brief, 250 *µ*L of the test sample was mixed with 385 *µ*L of 50 mM sodium phosphate buffer (pH = 7.5) and 35 *µ*L of enzyme solution (0.2 units/mL). After preincubation for 15 min at 37°C, 330 *µ*L of xanthine (150 *µ*M) was added as a reaction substrate, and after 15 min of incubation at 37°C, the absorbance of the reaction mixture was measured with a spectrophotometer at 295 nm. Allopurinol was used as a positive control (0.5; 1; 2.5; 5 *µ*g/mL). The inhibition percentage of each sample was calculated using the following formula:

(4) $$\begin{array}{c} PI\left(\%\right)=\left[\frac{\left(A-B\right)-\left(C-D\right)}{\left(A-B\right)}\right]\times 100\text{.} \end{array}$$

*A* is the absorbance of the enzyme with the substrate. *B* is the absorbance of the phosphate buffer solution with the substrate. *C* is the absorbance of the enzyme with the inhibitor and the substrate. *D* is the absorbance of the phosphate buffer solution with the substrate and the enzyme.

## 3. Results

### 3.1. Total Phenolics, Total Flavonoids, and Condensed Tannins Contents

Significant differences were noted in the parameters analyzed, except for the total flavonoid content where the *Napoleon* showed a high value compared to the other cultivars (Table 1). The total phenolic content ranged from 340 mg/g in *Napoleon* to 191 mg/g recorded in the *Burlat* stem extract. Compared to previous works, our result showed a higher amount of the total phenolic content [8, 11, 13, 14]. Interestingly, in another study focusing on fruit quality, the highest total phenolic content value was obtained for the *Napoleon* cultivar, with a value of 306.67 mg/100 g dry weight [5].

For flavonoids, the highest value recorded was for *Napoleon* with 34.31 mg/g. This observation is also true for tannins (9.25 mg/g). On the other hand, *Burlat* seems to be the poorest in all compounds, with 23.31 mg RE/g of flavonoids and no tannin detected. Furthermore, the amount of flavonoids is higher than those reported by some previously reported studies [8, 11, 14].

### 3.2. Antioxidant Activities

#### 3.2.1. DPPH Scavenging Activity

In this study, four cherry cultivars were evaluated. The free radical scavenging activity was expressed using IC_50_ values (concentration of the extract required to inhibit 50% of the initial DPPH free radical) (Table 2). The results showed that the percentage values of the free radical scavenging activity (% RSA) increased with increasing concentrations of the stem extracts. *Napoleon* extracts showed the most potent DPPH scavenging activity, while *Burlat* extracts showed the weakest activity. These results indicate that the level of phenolic compounds in the cultivars examined correlates with the values obtained by the DPPH test.

Additionally, all extracts exhibited a lower activity than the Trolox positive control (IC_50_ = 2.95 ± 0.12 *µ*g/mL) except for the *Napoleon* extract where the activity was the highest (IC_50_ = 2.51 ± 0.15 *µ*g/mL).

#### 3.2.2. Radical Cation ABTS^•+^ Scavenging Activity

As shown in Table 2, antioxidant activities by the DPPH method produce lower IC_50_ values (2.51 ± 0.15 to 7.78 ± 0.70 *µ*g/mL) than the ABTS assay, which appears to be a good method to express the antioxidant capacity of phenolic compounds in sweet cherry stems. IC_50_ values ranged from 55.38 ± 5.08 to 128.95 ± 16.18 *µ*g/mL, using the ABTS method (Table 2). All extracts exhibited lower activity than the Trolox positive control (IC_50_ = 30.86 ± 1.90 *µ*g/mL).

These results are consistent with those obtained for the phenolic compounds, flavonoids, condensed tannins contents, and DPPH values in which *Napoleon* has the highest dose of these compounds and explain the concentration related efficacy observed in the antioxidant experiments.

#### 3.2.3. Ferric Reducing Antioxidant Power (FRAP)

The presence of antioxidants in the samples would reduce Fe^3+^ to Fe^2+^ by donating an electron. The ferric reducing power of the samples is expressed as concentration equivalent *µ*g Trolox per gram of the extract, and the results are presented in Table 2. The reducing powers of the samples decreased as follows: *Van* (45.63) > *Coeur de pigeon* (35.13) > *Burlat* (34.31) > *Napoleon* (29.23) *µ*g TE/g of the extract.

### 3.3. Phytochemical Analysis by UHPLC-DAD/MS

**Figure 2:**
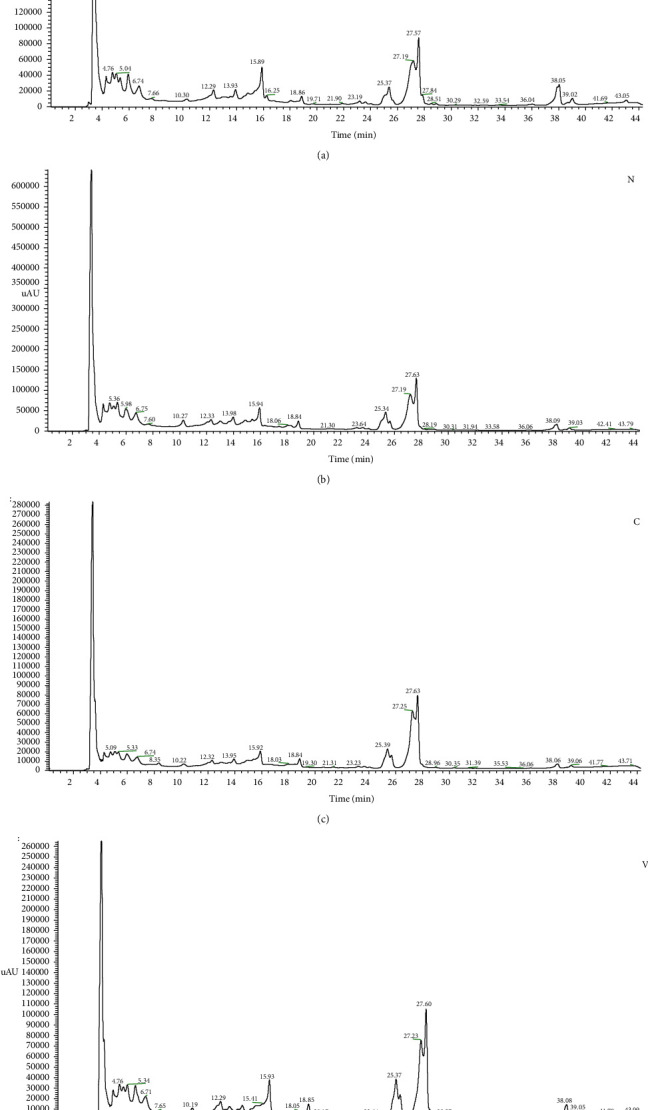
UHPLC chromatograms of phenolic compounds detected in sweet cherry stem's extracts recorded at 280 nm for different cultivars: (a) *Burlat* (B). (b) *Napoleon* (N). (c) *Coeur de pigeon* (C). (d) *Van* (V).

In this study, the analysis of phenolic compounds of cherry stems from four cultivars was performed by UHPLC-DAD/MS, and the chromatograms of each extract are shown in Figure 2. A significant difference was observed in the chromatograms of *Napoleon* cultivars compared to others. Additionally, regarding the high relative intensity of the peaks in most compounds detected in the *Napoleon* cultivar compared to the other cultivars and considering this semiquantitative method, this may explain the results obtained for *Napoleon* stem extracts in TPC, TFC, DPPH, and ABTS assays.

Tentative identification was suggested based on the information provided by the MS data and related information in the literature. The UHPLC-DAD/MS analysis of cherry stem extracts tentatively identified twenty-two compounds, which can be divided into five groups: six hydroxycinnamic acids (1, 3-4, 6, and 8-9), eight flavonols (2, 7, 10–13 and 16-17), one flavan-3-ols (5), five flavanones (15, 18–20 and 22), and two flavones (14 and 21).  Table 3 lists the tentatively identified phenolic compounds in the negative ionization mode, together with their retention time (min), their experimental *m*/*z* for the deprotonated molecular ion ([M-H]^−^), and their families.

The peak area of each compound was obtained from the UHPLC data, and the results obtained are presented in Table 4, together with the relative percentage of phenolic acids and flavonoids, which are also subdivided into the relative percentage of each of the four identified classes. Although it was not possible to quantify each phenolic compound individually due to the scarcity of standards, the relative percentage of each phenolic family (calculated based on the chromatographic peak areas) can provide us with an estimate of the relative abundance of each family of compounds in the sample. Nevertheless, it should be noted that this is a semiquantitative method, as compounds may exhibit different sensitivities in UHPLC-MS.

Table 4 aims to present the different concentrations of the different compounds identified. Generally, hydroxycinnamic acids were the only class of phenolic acids identified in all cultivars, with a high relative percentage of this class in *Napoleon* and *Burlat* cultivars. However, other works have identified hydroxybenzoic acids and their glucosides in addition to classes of hydroxycinnamic acids [10, 14].

Regarding the hydroxycinnamic acids, compounds 1, 3-4, 6, 8, and 9 were identified according to MS data and the previous studies [9, 10, 12–14].

**Figure 3:**
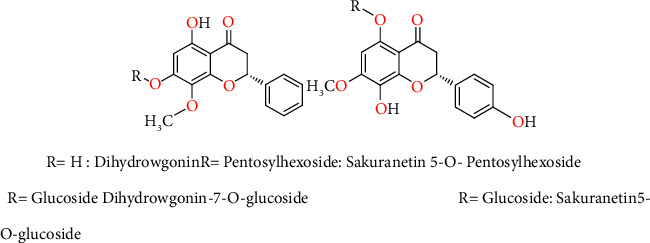
Chemical structure of sakuranetin and dihydrowgonin with their glucosides.

Finally, with the respect to the large family of flavonoids, two compounds were distinguished by the high relative intensity of their peak; compound 19 can be either dihydrowogonin or sakuranetin-O-pentosylhexoside and compound 20 may correspond to either dihydrowogonin 7-O-glucoside or sakuranetin 5-O-glucoside (Figure 3), as both compounds are described as being the main compounds detected in cherry stems, as also observed in this case [10, 12].

### 3.4. Assessment of Bioactive Properties

#### 3.4.1. *α*-Amylase Inhibitory Activity

In this study, the ability of the 80% methanol extract of sweet cherry stems from different cultivars to inhibit the *α*-amylase enzyme was investigated. Three of the analyzed extracts inhibited this enzyme at a dose of 1 mg/mL (Table 5).

The most active stem extracts were *Burlat* (85.57 ± 1.09%) and *Napoleon* (68.01 ± 3.52%) cultivars, while stem extract from *Coeur de pigeon* cultivar was the least active. These values are difficult to compare, given the lack of studies carried out in this field. However, the *α*-amylase inhibitory activity of stem extracts for *Burlat* and *Napoleon* cultivars can be considered comparable to that of green tea, oolong tea, and guava leaf extracts (% I = 21.0 ± 3.7, 10.9 ± 2.7, and 32.4 ± 9.5, respectively, at 250 *µ*g/mL) [24].

Our stem extracts showed significantly higher inhibition than those obtained with *Techlovan*, *Sumhit*, and *Rivan* sweet cherry extracts with IC_50_ values of 46.7 ± 0.4, 74.4 ± 1.6, and 78.3 ± 1.4 mg/mL, expressed as dried hydromethanolic and hydrochloric acid extracts, respectively) [25].

#### 3.4.2. Anti-Xanthine Oxidase Inhibitory Activity

XOD catalyses the transformation of purine bases into uric acid and H_2_O_2_. Underexcretion and/or overproduction of this acid lead to the incidence of hyperuricemia in the form of gout [26]. Consequently, the use of XOD inhibitors is considered as a hypouricemic treatment of gout by stopping the production of uric acid.

In this study, sweet cherry stem's extracts for all cultivars were tested for the XOD inhibitory activity at 1 mg/mL in the final reaction mixture. The results were expressed as inhibition percentage (%) and shown in Table 6. A more potent XOD activity of the extract is indicated by a higher percentage inhibition.

The inhibition percentages varied from 26.25 ± 0.90% to 40.63 ± 2.37% according to the cultivar. *Van* cultivar exhibited the highest activity (40.63 ± 2.37%) compared to the others. This result may be explained by the high relative percentage of flavonoids in *Van* cultivar (90.65% shown in Table 4). Flavonoids are antioxidants that inhibit XOD [27]. Additionally, all stem extracts exhibited a lower activity than the pure compound allopurinol which showed inhibitory effects of 20%, 44.38%, 89.11%, and 90.88% at 0.5, 1, 2.5, and 5 *µ*g/mL, respectively.

## 4. Discussion

### 4.1. Radical Cation ABTS^•+^ Scavenging Activity

The ABTS assay is an excellent approach to study the antioxidant activity of hydrogen‐donating agents and chain breakers. It is available for both hydrophilic and lipophilic antioxidant media; while the DPPH assay uses a radical dissolved in an organic medium and is, consequently, applicable to hydrophobic media [28].

Regarding the tests in which the total contents of phenolic compounds, flavonoids, and tannins were evaluated (Table 1), as well as for the method of antioxidant activities (Table 2), the results were similar to those obtained by the FRAP method, with the exception of the *Napoleon* extract. This may be because the compounds reacting with DPPH radicals may not be the same as those reacting with the TPTZ-Fe^3+^ complex. According to the DPPH method, the radical is neutralized when it receives H^+^ and/or electrons from the antioxidants, but for the FRAP assay, the TPTZ-Fe^3+^ complex was reduced to TPTZ-Fe^2+^ only by an electron transfer mechanism by compounds with a redox potential below 0.7 V [29, 30].

Additionally, the measure of reducing power seems to be related to the degree of hydroxylation of the benzene ring and its possible modification by secondary reactions as well as to the extent of conjugation to the phenolic compound [31].

In the present study, the reducing power ability of our extracts was found to be more potent than that revealed by the hydromethanolic stem extracts from sweet cherry cultivars, namely, *Burlat, Early Bigi NC, Lapins, and Van* (15.15 ± 1.40 *µ*g·TE/g, 18.15 ± 2.24 *µ*g·TE/g, 26.66 ± 2.26 *µ*g·TE/g, and 18.21 ± 2.19 *µ*g·TE/g extract, respectively) [14].

### 4.2. Phytochemical Analysis by UHPLC-DAD/MS

The analysis of phenolic compounds is of great interest to scientists, manufacturers, and consumers for their impact on product quality and for their protective and preventive functions in the pathogenesis of certain types of cancer and several other chronic diseases. There are many reports in the literature on the identification and quantification of phenolic compounds in *Prunus avium* (L.) fruits. The most commonly identified compounds are phenolic acids (neochlorogenic, chlorogenic, and *p*-coumaroylquinic acids), anthocyanins, flavonols (rutin), and flavan-3-ols (catechin and epicatechin) [10, 16, 31, 32].

To date, few publications have been devoted to the chemical composition of sweet cherry stem. Bursal et al. (2013) studied phenolic acids in ethanolic and aqueous extracts of cherry stems by LC-MS/MS [11]. They have identified pyrogallol, ferulic acid, *p*-coumaric acid, gallic acid, *p*-glucosidic acid, ascorbic acid, and *p*-hydroxybenzoic acid. Bastos et al. compared the HPLC phenolic profile of fruits and stems of *P. avium* L. They detected more phenolic compounds in the stem than in the fruit [10]. Ademović et al. identified phenolic compounds in the alcoholic and aqueous extracts of wild cherry stem [8]. They found that quercetin and (+)-catechin were the two main compounds detected in the alcoholic extract, followed by chlorogenic acid and rutin. Aires et al. analyzed glycosylated flavonoids extracted from sweet cherry stems [9]. They found a high content of sakuranetin, ferulic acid, *p*-coumaric acid, *p*-coumaroylquinic acid, chlorogenic acid, and its isomer neochlorogenic acid. In 2020, Peixoto et al. explored the phenolic profile of infusions prepared with four distinct commercial brands of cherry stems (*Prunus avium* L.) by UHPLC-q-TOF-MS/MS, and they identified eight distinct classes of phenolic compounds [13]. To our knowledge, only one publication has reported the phenolic composition by HPLC-DAD of stem extracts from four sweet cherry cultivars, with the identification of seventeen compounds [14].

### 4.3. Assessment of Bioactive Properties

#### 4.3.1. *α*-Amylase Inhibitory Activity

Diabetes mellitus type 2 (DMT2) is a common chronic metabolic disease. It is caused by abnormalities in carbohydrate metabolism associated with low blood insulin levels or impaired sensitivity of target organs to insulin. The *α*-amylase enzyme is heavily involved in the digestion of carbohydrates. Therefore, its inhibition will be a contemporary therapeutic approach of DMT2 [33]. To the best of our knowledge, this is the first report on the inhibitory effect of sweet cherry stem extracts from different cultivars against the *α*-amylase enzyme. However, sweet cherries have already been shown to be a potential inhibitor of this enzyme, mainly due to their high content of phenolic compounds [7, 25]. In the current study, the *α*-amylase inhibitory activity was not correlated with total phenolic compounds (expressed as % of the peak area in Table 4) in the analyzed extracts; however, some subclasses of the phenolic compound were correlated with this activity, especially for *Burlat* cultivar with a high estimate of the relative abundance of flavan-3-ol (catechin) (9.32%) compared to the other cultivars. This correlation was still not satisfactory. Some poor correlations have also been reported previously [34, 35]. They considered that the specific type (number, type, and position of OH groups) of phenolic compounds is more significant for inhibitory effects on digestive enzymes than the total amounts of phenolic compounds.

#### 4.3.2. Anti-Xanthine Oxidase Inhibitory Activity

XOD catalyses the transformation of purine bases into uric acid and H_2_O_2_. Underexcretion and/or overproduction of this acid lead to the incidence of hyperuricemia in the form of gout [26]. Consequently, the use of XOD inhibitors is considered as a hypouricemic treatment of gout by stopping the production of uric acid. No previous research has been conducted on the inhibitory activity of the sweet cherry stem extract against XOD; however, some studies have shown that the consumption of sweet cherries reduces serum urate levels in healthy women and suggests a potential application of cherries for the treatment of gout [24, 36, 37].

## 5. Conclusion

This study was conducted on the stem of four sweet cherry cultivars. The results showed that the extracts obtained from this plant material are rich in polyphenols with a content of 340 ± 12.06, 244 ± 10.20, 232 ± 5.07, and 191 ± 3.10 mg GAE/g extract for the *Napoleon*, *Coeur de pigeon*, *Van*, and *Burlat* cultivars, respectively. In terms of flavonoids and tannins, again, the *Napoleon* cultivar was the most abundant with values of 34.31 ± 2.08 mg RE/g extract and 9.25 ± 1.50 mg CE/g extract, while *Burlat* was low in tannins. These results explain the low IC_50_ values observed for *Napoleon* (2.51 *µ*g/mL for DPPH and 55.38 *µ*g/mL for ABTS) close to those obtained by Trolox. The UHPLC-DAD/MS analysis of cherry stem extracts resulted in the tentative identification of twenty-two compounds belonging to five distinct groups. The *Napoleon* cultivar had the highest amount of all compounds based on their high peak intensity. The major phenolic compounds identified in all cultivars were sakuranetin and dihydrowgonin with their glucosides. Only stem extracts from *Burlat* and *Napoleon* cultivars were able to inhibit the *α*-amylase enzyme with values of 85.57 ± 1.09% and 68.01 ± 3.52%, respectively. Some subclasses of the phenolic compounds correlated with this activity, especially for *Burlat* cultivar with a high estimated relative abundance of flavan-3-ols (catechin) (9.32%) compared to the other cultivars. All stem extracts have proved their ability to inhibit the XOD enzyme with a high value for *Van* cultivar 40.63 ± 2.37%. This work shows that the treatment of certain diseases, such as DMT2 and gout, can be based on the choice of some stem cultivars. However, these stems need to be more investigated, and further studies are required to explore nutraceutical and pharmacological formulations or antioxidant preservatives for the food industry and their impacts on human health.

## Figures and Tables

**Table 1:** Total phenolic, total flavonoid, and condensed tannins contents of the studied cherry stem extracts.

Cultivars	TPC (mg GAE/g extract)	TFC (mg RE/g extract)	TTC (mg CE/g extract)
*Burlat*	191 ± 3.10	23.31 ± 0.90	—
*Napoleon*	340 ± 12.06	34.31 ± 2.08	9.25 ± 1.50
*Coeur de pigeon*	244 ± 10.20	23.75 ± 1.02	1.13 ± 0.90
*Van*	232 ± 5.07	24.37 ± 1.20	4.07 ± 1.28

**Table 2:** IC_50_ values of the DPPH, ABTS, and FRAP tests for the different extracts.

Cultivars	DPPH (IC_50_ (*μ*g/mL))	ABTS (IC_50_ (*μ*g/mL))	FRAP (*μ*g·TE/g extract)
*Burlat*	7.78 ± 0.70	128.95 ± 16.18	34.34 ± 1.17
*Napoleon*	2.51 ± 0.15	55.38 ± 5.08	29.23 ± 2.91
*Coeur de pigeon*	5.09 ± 1.02	126.26 ± 14.37	35.13 ± 1.09
*Van*	4.77 ± 0.80	85.4 ± 8.76	45.63 ± 4.20
*Trolox*	2.95 ± 0.12	30.86 ± 1.90	—

**Table 3:** Main phenolic compounds identified by UHPLC-MS in cherry stem's extracts of four cultivars: *Burlat* (B), *Napoleaon* (N), *Coeur de pigeon* (C), and *Van* (V).

Peaks	Retention times (min)				Molecular ions	Family	Compounds
	B	N	C	V	[M-H]^−^(*m/z*)		
1	4.29	4.30	4.26	4.29	353	Hydroxycinnamic acid	3-O-caffeoylquinic acid
2	4.76	4.78	4.75	4.76	465	Flavonol	Taxifolin-7-O-hexoside
3	5.04	5.09	5.09	5.05	337	Hydroxycinnamic acid	p-Coumaroyl quinic acid
4	ni	5.36	5.33	ni	341	Hydroxycinnamic acid	Caffeic acid hexoside
5	5.93	5.9	5.98	5.94	289	Flavan-3-ols	Catechin
6	6.74ni	6.75	6,74	ni	179	Hydroxycinnamic acid	Caffeic acid
7	7.66	7.60	8.35	ni	449	Flavonol	Aromadendrin-7-O-hexoside
8	10,3	10.27	10.22	10.19	325	Hydroxycinnamic acid	*p-*coumaricacidhexoside
9	12.29	12.33	12.31	12.29	355	Hydroxycinnamic acid	Ferulic acid hexoside
10	ni	13.02	ni	ni	449	Flavonol	Aromadendrin-O-hexoside
11	13.93	13.98	13.95	13.91	463	Flavonol	Methyl-aromadendrin-O-hexoside
12	14.86	14.90	14.99	14.92	609	Flavonol	Quercetin-3-O-rutinoside
13	15.89	15.94	ni	15.93	463	Flavonol	Quercetin-3-O-glucoside
14	ni	18.06	18.03	18.05	431	Flavone	Genistein-7-O-glucoside
15	18.86	18.84	18.48	18.85	433	Flavanone	Naringenin-7-O-glucoside
16	ni	23.21	23.23	23.20	593	Flavonol	Kaempferol-3-O-rutinoside
17	23.61	23.64	23.66	23.64	447	Flavonol	Kaempferol-3-O-glucoside
18	25.37	25.34	25.39	25.37	549	Flavanone	Pinocembrin-O-pentosyl-hexoside
19	27.19	27.19	27.25	27.23	579	Flavanone	Dihydrowogonin/sakuranetin-O-pentosylhexoside
20	27.57	27.63	27.63	27.60	447	Flavanone	Dihydrowogonin7-O-glucoside/sakuranetin5-O-glucoside
21	38.05	38.09	38.06	38.07	415	Flavone	Chrysin-7-O-glucoside
22	39.02	39.03	ni	39.05	285	Flavanone	Dihydroxymethoxyflavanone

ni: not identified.

**Table 4:** Peak areas (^*∗*^10^4^) and average of each family obtained by HPLC/MS analysis of different cultivars: *Burlat* (B), *Napoleaon* (N), *Coeur de pigeon* (C), and *Van* (V).

Peaks	Compounds	Peak areas			
		B	N	C	V
1	3-O-caffeoylquinic acid	72.4	130.9	11.6	11.4
2	Taxifolin-7-O-hexoside	71.2	115.5	14.6	17.01
3	*p-*coumaroyl quinic acid	64	86.2	16.9	16.7
4	Caffeic acid hexoside	—	140.1	18.3	—
5	Catechin	91.5	128.7	22.01	35.02
6	Caffeic acid	64.8	109.5	18.2	—
7	Aromadendrin-7-O-hexoside	9.4	14.06	4.5	—
8	*p-*coumaric acid hexoside	4.7	27.9	4.8	11.1
9	Ferulic acid hexoside	29.9	28.4	7.2	22.5
10	Aromadendrin-O-hexoside	—	40.7	—	—
11	Methyl-aromadendrin-O-hexoside	14.6	42.2	6.2	6.2
12	Quercetin-3-O-rutinoside	20.4	48.9	2	1.1
13	Quercetin-3-O-glucoside	77.8	77.5	—	44.2
14	Genistein-7-O-glucoside	—	4.8	1.3	7.8
15	Naringenin-7-O-glucoside	10.9	23.5	1.8	19.2
16	Kaempferol-3-O-rutinoside	—	7.3	1.5	1.6
17	Kaempferol-3-O-glucoside	3.3	7.3	1.2	1.7
18	Pinocembrin-O-pentosyl-hexoside	71.4	113.3	45.9	84.9
19	Dihydrowogonin/sakuranetin 5-O-pentosylhexoside	206.3	278.3	170.9	188.3
20	Dihydrowogonin 7-O-glucoside/sakuranetin 5-O-glucoside	126.02	196.6	103.9	154.8
21	Chrysin-7-O-glucoside	28.9	36.3	6.8	27.8
22	Dihydroxy methoxyflavanone	13.4	8.1	—	8.4
	Total peak areas	980.92	1666.06	459.61	659.73
	% phenolic acids	24.04	31.39	16.75	9.35
	% hydroxycinnamic acid	24.04	31.39	16.75	9.35
	% flavonoids	75.96	68.61	83.25	90.65
	% flavan-3-ol	9.32	7.73	4.79	5.31
	% flavonol	20.06	21.21	6.53	10.89
	% flavanone	43.64	37.21	70.17	69.06
	% flavone	2.94	2.46	1.76	5.39

**Table 5:** Enzymatic in vitro *α*-amylase inhibition of hyperglycaemic of sweet cherry stem extracts from four cultivars.

	*Burlat*	*Napoleon*	*Coeur de pigeon*	*Van*
% Inhibition	85.57 ± 1.09	68.01 ± 3.52	3.87 ± 0.18	—
mg EAC/g of extract	190.40	143.17	29.38	—

**Table 6:** Xanthine oxidase inhibitory activity of sweet cherry stem extracts from four cultivars.

	*Burlat*	*Napoleon*	*Coeur de pigeon*	*Van*
PI (%)	27.94 ± 0.09	30.70 ± 1.80	26.25 ± 0.90	40.63 ± 2.37

## Data Availability

The data used to support the findings of this study are included within the article.
